# Speech-mediated manipulation of da Vinci surgical system for continuous surgical flow

**DOI:** 10.1007/s13534-024-00429-5

**Published:** 2024-10-12

**Authors:** Young Gyun Kim, Jae Woo Shim, Geunwu Gimm, Seongjoon Kang, Wounsuk Rhee, Jong Hyeon Lee, Byeong Soo Kim, Dan Yoon, Myungjoon Kim, Minwoo Cho, Sungwan Kim

**Affiliations:** 1 Interdisciplinary Program in Bioengineering, Seoul National University, 1 Gwanak-ro, Gwanak-gu, Seoul, 08826 Republic of Korea; 2 Department of Biomedical Engineering, Seoul National University College of Medicine, 103 Daehak-ro, Jongno- gu, Seoul, 03080 Republic of Korea; 3 Seoul National University Hospital, 101 Daehak-ro, Jongno-gu, Seoul, 03080 Republic of Korea; 4 MedInTech Inc., 60 Daehak-ro, Jongno-gu, Seoul, 03100 Republic of Korea; 5 Department of Transdisciplinary Medicine, Seoul National University Hospital, 101 Daehak-ro, Jongno-gu, Seoul, 03080 Republic of Korea; 6 Department of Medicine, Seoul National University College of Medicine, 103 Daehak-ro, Jongno-gu, Seoul, 03080 Republic of Korea; 7 Artificial Intelligence Institute, Seoul National University, 1 Gwanak-ro, Gwanak-gu, Seoul, 08826 Republic of Korea

**Keywords:** da Vinci research kit, da Vinci surgical system, Ergonomics, Human-robot interaction, Speech recognition, Usability

## Abstract

With the advent of robot-assisted surgery, user-friendly technologies have been applied to the da Vinci surgical system (dVSS), and their efficacy has been validated in worldwide surgical fields. However, further improvements are required to the traditional manipulation methods, which cannot control an endoscope and surgical instruments simultaneously. This study proposes a speech recognition control interface (SRCI) for controlling the endoscope via speech commands while manipulating surgical instruments to replace the traditional method. The usability-focused comparisons of the newly proposed SRCI-based and the traditional manipulation method were conducted based on ISO 9241-11. 20 surgeons and 18 novices evaluated both manipulation methods through the line tracking task (LTT) and sea spike pod task (SSPT). After the tasks, they responded to the globally reliable questionnaires: after-scenario questionnaire (ASQ), system usability scale (SUS), and NASA task load index (TLX). The completion times in the LTT and SSPT using the proposed method were 44.72% and 26.59% respectively less than the traditional method, which shows statistically significant differences (*p* < 0.001). The overall results of ASQ, SUS, and NASA TLX were positive for the proposed method, especially substantial reductions in the workloads such as physical demands and efforts (*p* < 0.05). The proposed speech-mediated method can be a candidate suitable for the simultaneous manipulation of an endoscope and surgical instruments in dVSS-used robotic surgery. Therefore, it can replace the traditional method when controlling the endoscope while manipulating the surgical instruments, which contributes to enabling the continuous surgical flow in operations consequentially.

**Supplementary Information:**

The online version contains supplementary material available at 10.1007/s13534-024-00429-5.

## Introduction

Since the late 1990s, research on minimally invasive surgery (MIS) has been continuing owing to its clinical significance. Consequently, it has been widely adopted in the medical field for various applications, such as urology, gynecology, general surgery, and so on [1]. Compared to open surgery, MIS provides more advantages, such as smaller incisions, shorter hospital stays, and a lower risk of postoperative complications [2, 3]. However, several significant issues persist, including prolonged operation time due to limited degrees of freedom (DOFs) of surgical instruments in the restricted workspace of the body, extended learning curve compared to that for the traditional counterpart, and poor hand–eye coordination [4]. To overcome these drawbacks, robot-assisted surgery (RAS) has been introduced, user-friendly technologies such as a high-definition three-dimensional (3D) endoscope system have been grafted, and higher DOFs have been achieved through the incorporation of additional joints, motion scaling, and hand tremor compensation [5]. One of the representative RAS platforms, the da Vinci surgical system (dVSS, Intuitive Surgical, Inc., Sunnyvale, California, USA), allows surgeons to perform surgery in an ergonomic operative environment with high-quality 3D visualization, hand tremor stabilization, and improved surgical instruments [5, 6]. Since the introduction of the dVSS, more exquisite and safe surgeries have become possible, and its efficacy has been validated globally in various surgical fields [1, 5–7].

The dVSS is a primary–secondary system in which the commands operated by the surgeon in the primary part are interlocked with the secondary part, reflecting the surgeon’s intentions in real-time [1]. The primary system, namely the surgeon console, functions as the surgeon’s main workspace and is comprised of a stereo viewer, two master tool manipulators (MTMs), and a foot pedal tray. The secondary system, namely the patient side cart, has three patient side manipulators (PSMs) and one endoscope camera manipulator (ECM) for performing invasive surgical operations and observing surgical sites, respectively. However, to manipulate the multiple PSMs and ECM with two MTMs, the surgeons should push the foot pedal to switch the access toward PSMs or ECM during the operation and then control the corresponding manipulators, resulting in issues such as long operation times and cognitive loads [8]. Therefore, to control the ECM while manipulating the PSMs with the MTMs, various concepts, such as using the head motion, foot, and fingers that remain unused when handling clutches on the MTMs, as well as speech recognition technology, have been proposed [9–13].

Among the candidates, speech recognition is considered as one of the most natural and intuitive interfaces for surgeon-to-robot communication [12, 13]. Moreover, this technology has been rapidly improved with the development of machine learning algorithms and has been adopted in various applications, including personal computers, smartphones, and surgical robots [13, 14]. For human–robot interactions, speech recognition is more efficient than various hand gestures or other user movements, and multiple studies have proposed speech recognition as a promising tool for improving surgical robots [15–17]. In addition, due to its intuitive nature, more surgical instruments can be employed without requiring complex manipulations with arms or legs [18, 19]. Notably, 87.9% of clinicians have an optimistic view of automatic speech recognition technology in the operating room and highly value its future potential [20]. Considering the diverse advantages and contributions of the speech-mediated methods, a speech recognition control interface (SRCI) has been proposed herein for controlling the ECM through speech commands while manipulating the surgical instruments installed on the PSMs.

The present study set the hypothesis that the proposed methods can improve the discontinuous surgical flow when the surgeons uses the dVSS. Therefore, the usability of the SRCI has been evaluated and compared with that of the traditional method as a control, which manipulates the ECM with MTMs in the surgeon console. To investigate the replaceability of the MTMs-based method to control the ECM, experimental environments have been established based on the da Vinci research kit (dVRK, Intuitive Surgical, Inc., Sunnyvale, California, USA), which has the same manipulation mechanism as the dVSS and utilized as a baseline for various user evaluations about dVSS [1, 9–11, 21–23]. It contributes to novelties in that the participants including the surgeons conduct a series of scenarios by themselves under the identical condition of the manipulation for the actual dVSS-used environment, unlikely in the previous studies just applying the speech recognition technology to the surgical robot [16–19, 24–26]. Therefore, the present study ergonomically validated the feasibility of speech-mediated manipulation to replace the traditional method using MTMs for resolving the discontinuous surgical flow in the operation.

## Materials and methods

### Setup

#### dVRK

**Fig. 1:**
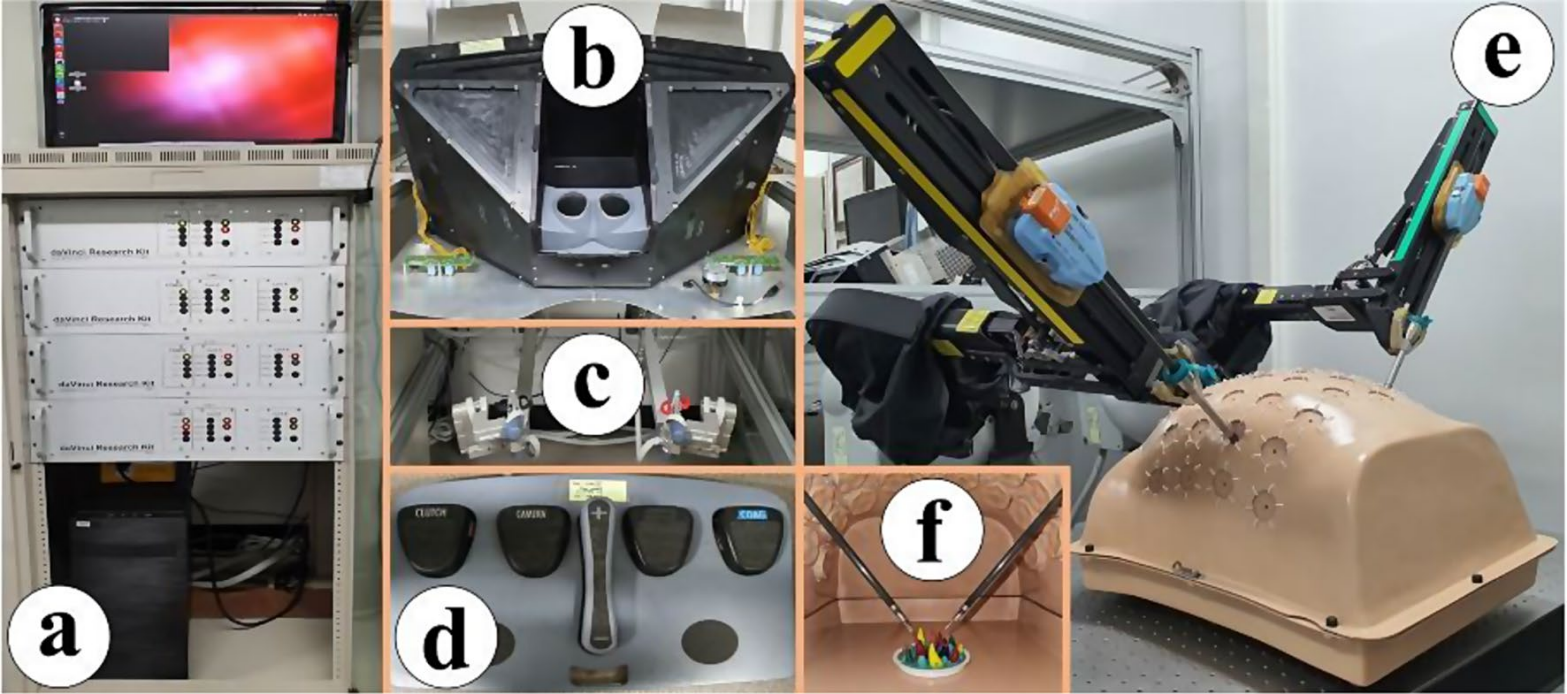
dVRK: **a** 8-axis motor control units, **b** stereo viewer, **c** MTMs, **d** foot pedal tray, **e** PSMs, **f** sea spikes pod

The dVRK, donated by Intuitive Surgical Inc. in 2014, is the main platform for research on ergonomic improvements of the dVSS [1]. It comprises four 8-axis motor control units, a stereo viewer, two MTMs, two PSMs, and a foot pedal tray, as demonstrated in Fig. 1. The dVRK is configured using an open-source robot operating system framework and libraries developed by Johns Hopkins University [27, 28]. The methods for manipulating the dVRK are as follows: The operation of the MTMs, which are the master controllers for the PSMs, is remotely interlocked with the execution of each of the PSMs. In detail, the movements of the MTMs are directly transferred to the PSMs, and the actions of the surgical instruments installed on the PSMs, such as gripping motion, can be controlled by switching the status of engagement or disengagement of finger clutches at the MTMs in real-time. The scale factor that determines the moving ratio of the PSM according to the MTM is preset by the dVRK software to simulate the operation environment [29]. Therefore, the user can control the PSMs with MTMs remotely viewing the stereo viewer in real-time.

#### EECS

**Fig. 2:**
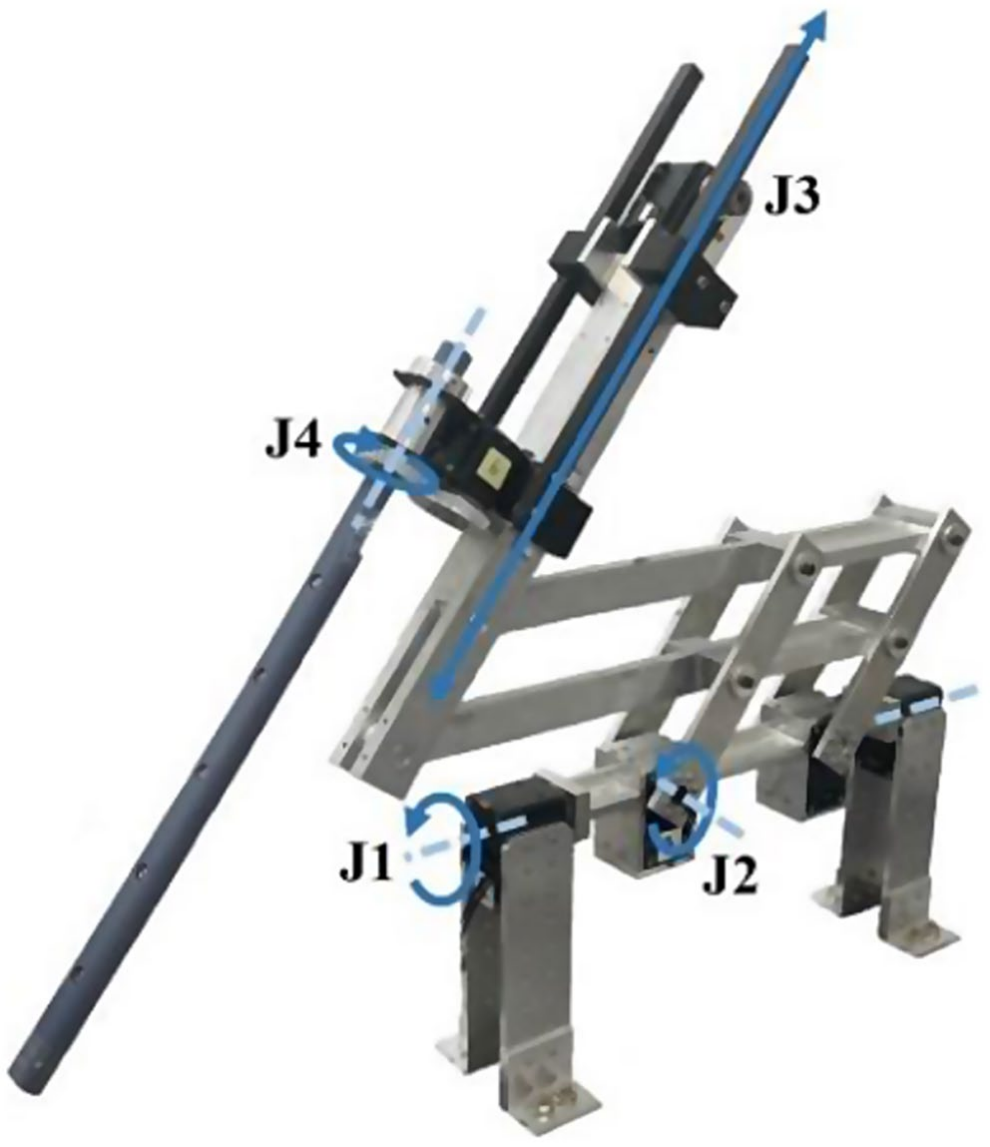
EECS

Because the ECM is not included as a basic component of the dVRK, an enhanced endoscope control system (EECS) has been developed to obtain stereoscopic images from the test bed and imitate the motion of the ECM as illustrated in Fig. 2. The EECS, which has a double-parallelogram structure to enable fulcrum point motions, can provide various operations including roll, pitch, insertion, and rotation movements, using six motors that are controlled using the Dynamixel Wizard software [9]. In detail, *J1* (Motors 1 and 2) is for roll, *J2* (Motors 3 and 4) is for pitch, *J3* (Motor 5) is for insertion, and *J4* (Motor 6) is for rotation movements. The technical specifications of the EECS are represented in Table 1. Further, the moving ratio of the EECS is preset to provide analogous movements of the ECM. Two camera modules with a maximum resolution of 1,920$\:\:\times\:\:$1,080 pixels are installed to capture the images in real-time. To facilitate 3D visualization to the stereo viewer of the dVRK, a rectification process based on Unity and Visual Studio 2019 is implemented. The users can see the stereoscopic images from the EECS through the stereo viewer.

**Table 1:** Technical specifications of EECS

Specification items	Unit	Joints	
Installing Posture			Floor mounted
Construction			Two-parallel link
Degrees of freedom			4
Operational range	degree	J1	± 60
		J2	± 60
		J4	± 90
	mm	J3	160
Maximum speed	rpm	J1, J2	29.31
	rpm	J4	52.67
	mm/s	J3	44.00
Resolution	deg/pulse	J1, J2, J4	0.0879
	pulse/rev	J1, J2, J4	4,096
	/mm	J3	0.00002114

#### SRCI

**Fig. 3:**
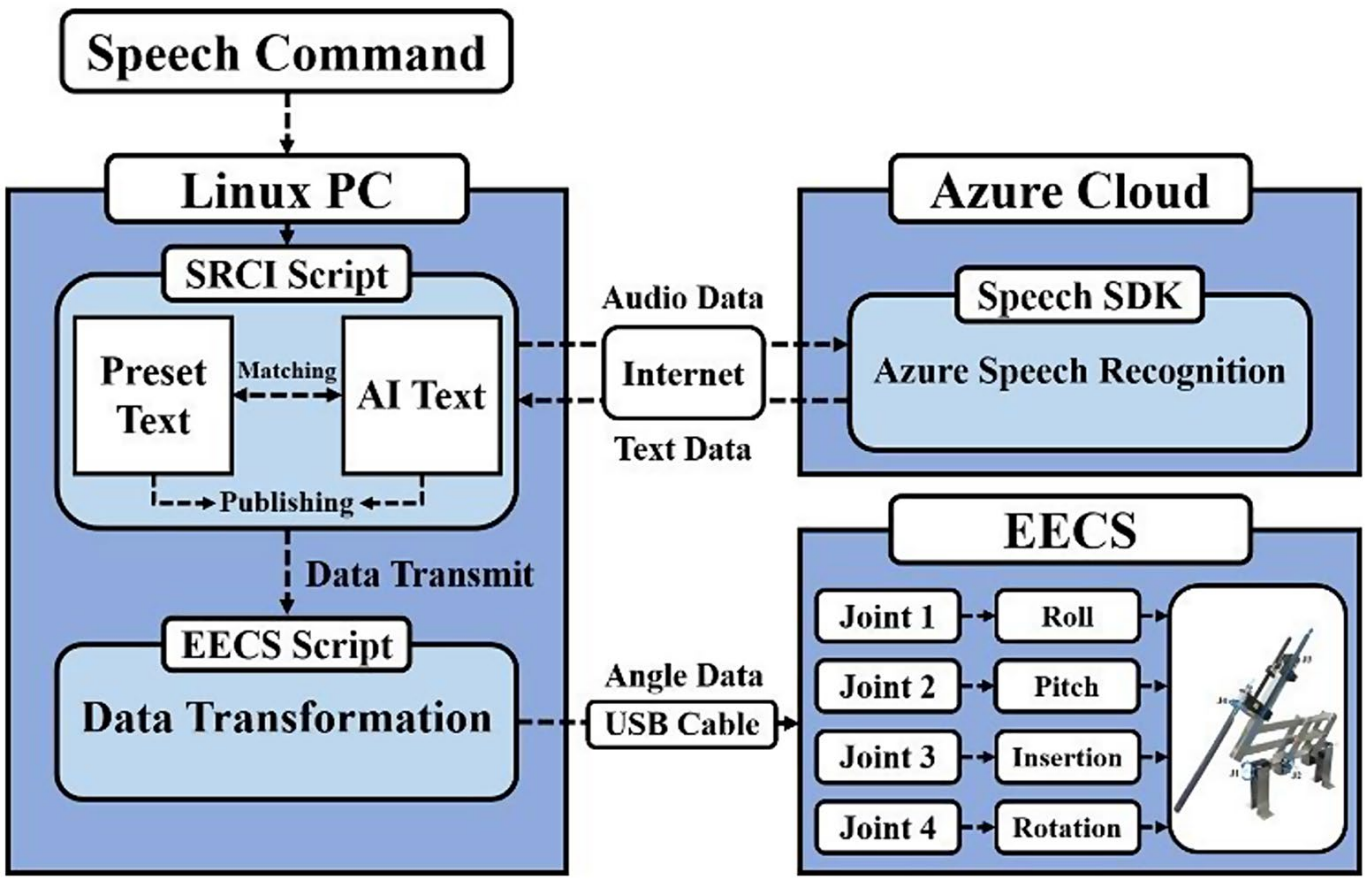
Diagram of SRCI

The SRCI was designed by the Microsoft Azure model to incorporate speech recognition technology, as illustrated in Fig. 3 [30]. When a user’s speech commands are input to the Linux PC, this speech command converts audio data into text data through the speech SDK in Azure Cloud. In the present study, rule-based commands, including “Endo up,” “Endo down,” “Endo left,” “Endo right,” “Endo in,” “Endo out,” and “Endo stop,” have been implemented. The prefix “Endo”, inspired by the word “endoscope”, is set to minimize the false positive rate induced by ordinary conversations. “Endo in” and “Endo out” are the commands for zooming in and out, respectively. If the user’s speech commands are interpreted to one of the predefined text-type commands by the sequence matching formula, then the corresponding function is executed, and the EECS continuously moves in a specific direction [31]. Because the EECS was designed to mimic the operational mechanism of ECM, therefore, all commands in the present study can control all movements of the EECS. From the perspective of patient safety, the EECS has policies that operate at the preset moving ratio in the range of the maximum speed and stop the movement automatically if it moves over its workspace limitation.

$$D_{r}o=(2*k_{m})/(|S_{1}|+|S_{2}|)$$

### Manipulation methods

#### Traditional method

To control the surgical instruments installed on the respective PSMs with MTMs, a user manipulates the MTMs based on their operation intentions while continuously pushing a “coag” button at the right side of the foot pedal tray. If the working range of the instruments on the patient side is limited during the operation, then the user should manipulate the MTMs while continuously pushing a “clutch” button at the left side on the foot pedal tray. Because the movements of the MTMs are not interconnected to the instruments while pushing a “clutch” button, a user can readjust the position of the MTMs to prepare to control the instruments again. Likewise, the EECS can be controlled by manipulating the MTMs with pedals.

#### Proposed method

The manipulation methods of the surgical instruments with the MTMs and pedals are the same as the traditional methods. To control the EECS, a user speaks the rule-based commands to the microphone installed on the stereo viewer. Then, the EECS continuously moves based on the user’s intended direction. In general, a user can speak the “Endo stop” command to stop the control of the EECS, and it can be applied to abnormal situations in which the SRCI does not recognize or misinterpret the commands. Technically, the diagonal movements of the EECS are possible, however, those are restricted to minimize the tendency difference from person to person in the present study. Considering the safety issue, the EECS was designed thoroughly to be controlled in the inner range of the workspace simulated in real robotic surgery.

### Participants

Following the approval of the institutional review board (IRB) of Seoul National University Hospital (IRB No: H-2107-167-1236), a total of 38 Korean participants (age: 30.16 ± 4.09) were randomly recruited based on previous studies [22, 23]. To investigate whether the difference in task performance or usability between the traditional and proposed methods varies with the level of expertise, both the surgeon group and novice group that represents the extreme end of inexperienced surgeons were recruited. The surgeon group comprised 15 fellows (age: 34.27 ± 2.68) and 5 residents (age: 30.40 ± 2.05) from gastrointestinal, coloanal, endocrine, ophthalmologic, and orthopedic surgery fields. The novice group included 18 novices (age: 26.67 ± 0.97), who were not experts in the medical field. In the surgeon group, only male participants were recruited, therefore, the sex composition of all participants was 32 males (age: 30.91 ± 4.07) and 6 females (age: 26.17 ± 1.03). Before the usability evaluations, instructions and cautions to operate the dVRK, based on the traditional and proposed methods, were provided enough to all the participants for safe manipulation.

### Procedure of usability evaluation

To investigate the replaceability from MTM to SRCI based on ISO 9241-11 [32], the usability of traditional and proposed methods has been evaluated in analogous environments using the dVSS. In detail, the participants perform tasks using the dVRK and respond to various questionnaires based on their experience of using both these methods. The tasks are designed based on previous studies, and globally reliable questionnaires focused on usability are selected [32–37]. The user evaluation conducted in the present study is characterized by a within-subject design, in which all the participants conduct identical tasks using the traditional and proposed methods. To minimize issues related to learnability, initially, half of the participants performed the evaluation using the traditional method, while the remaining participants performed the tasks using the proposed method.

### Task performance

#### LTT

**Fig. 4:**
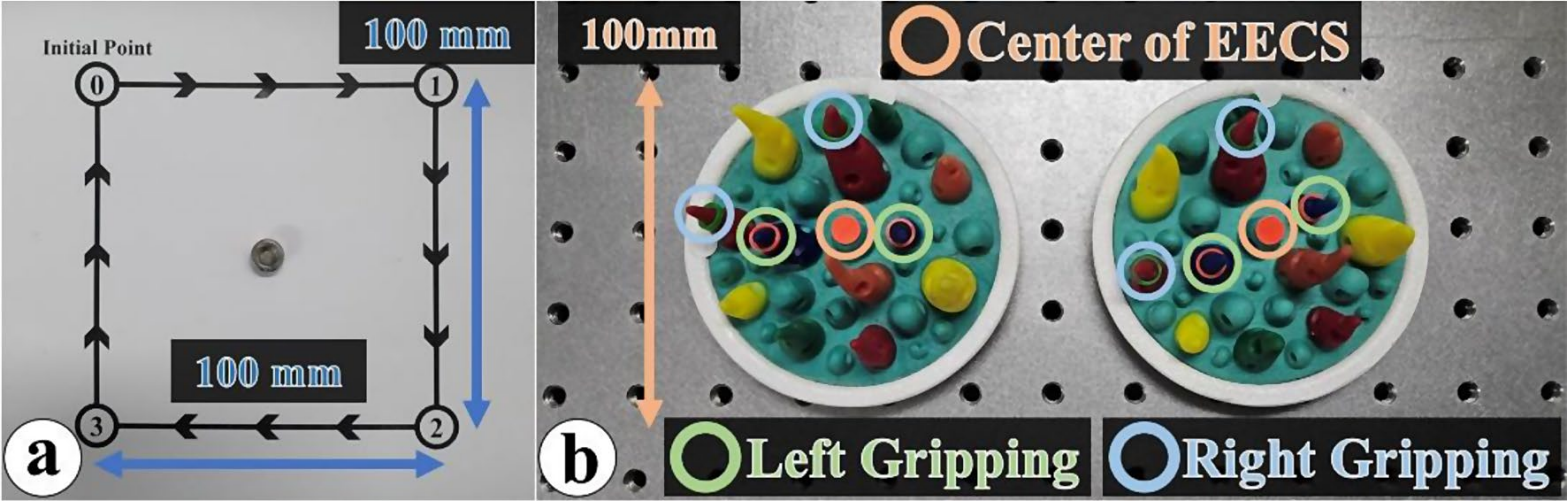
Usability evaluation: **a** LTT, **b** SSPT

In the line tracking task (LTT), which has been utilized in a variety of types in previous studies, the participants move the EECS and surgical instruments together by following the arrows in the sequence of 0, 1, 2, 3, and 0, as illustrated in Fig. 4a [11, 38, 39]. The shape of the route to be tracked is a square, and each line to be tracked is of length 0.1 m, thus, a total distance of 0.4 m is covered, which is the validated space in the abdomen model [38]. At each point with a designated number, the participants perform the gripping motion once with the surgical instruments. All the participants perform the LTT twice, and the total time required to move the total distance is measured and compared for both the traditional and proposed methods. The mean time and standard deviation (SD) are determined to compare and analyze the results obtained from both groups for the two methods, as in the previous studies [21–23].

#### SSPT

As an application, the sea spikes pods task (SSPT) is designed based on the sea spike pods that are utilized by medical interns to exercise the dVSS and can be modified for diverse purposes [23, 40, 41]. In the present study, the participants control the EECS and surgical instruments to change the visual field and grip the designated spikes on the respective sea spike pods, as shown in Fig. 4b. The detailed steps are as follows: The participants grip the identical colored spikes with right and left instruments one at the same time, and then move the visual field with the EECS to the opposite sea spike pod. At the opposite sea spike pod, the participants perform the same actions and then return to the original sea spike pod. All the participants perform the SSPT twice, and the corresponding completion times are measured, referring to [21–23].

### Questionnaires

#### ASQ

The after-scenario questionnaire (ASQ), which indicates 0.96 global reliability (Cronbach alpha score) ranging from 0 (completely unreliable) to 1 (perfectly reliable), is a survey used immediately following scenario completion in scenario-based usability studies [33–35]. As the user evaluation in the present study includes LTTs and SSPTs, which have protocol scenarios, the ASQ survey is implemented to examine the satisfaction of the participants. The ASQ has three questions, including comments, and uses a seven-point Likert scale ranging from + 1 (strongly agree) to + 7 (strongly disagree), as presented in Table 2 [34].

**Table 2:** ASQ (global reliability: 0.96)

No.	Question	Score*						
		1	2	3	4	5	6	7
1	Overall, I am satisfied with the ease of completing the tasks in this scenario.	○	○	○	○	○	○	○
2	Overall, I am satisfied with the amount of time it took to complete the tasks in this scenario.	○	○	○	○	○	○	○
3	Overall, I am satisfied with the support information when completing the tasks.	○	○	○	○	○	○	○

*Seven-point Likert scale ranging from + 1 (strongly agree) to + 7 (strongly disagree)

#### SUS

The system usability scale (SUS) survey usually reflects a user’s subjective rating of usability rapidly and easily [36, 37]. The SUS has 10 questions and uses a five-point Likert scale ranging from + 1 (strongly disagree) to + 5 (strongly agree), as presented in Table 3. The questions are categorized as positive and negative attributes, and comprehensive scores can be calculated using the given formula to be considered as adjective ratings: best imaginable, excellent, good, OK, poor, and worst imaginable [36, 37].

**Table 3:** SUS (global reliability: 0.92)

No.	Question	Score*				
		1	2	3	4	5
1	I think that I would like to use this system frequently.	○	○	○	○	○
2	I found the system unnecessarily complex.	○	○	○	○	○
3	I thought the system was easy to use.	○	○	○	○	○
4	I think that I would need the support of a technical person to be able to this system.	○	○	○	○	○
5	I found the various functions in this system were well integrated.	○	○	○	○	○
6	I thought there was too much inconsistency in this system.	○	○	○	○	○
7	I would imagine that most people would learn to use this system very quickly.	○	○	○	○	○
8	I found the system very awkward to use.	○	○	○	○	○
9	I felt very confident using the system.	○	○	○	○	○
10	I needed to learn a lot of things before I could get going with the system.	○	○	○	○	○

*Five-point Likert scale ranging from + 1 (strongly disagree) to + 5 (strongly agree)

#### NASA TLX

**Fig. 5:**
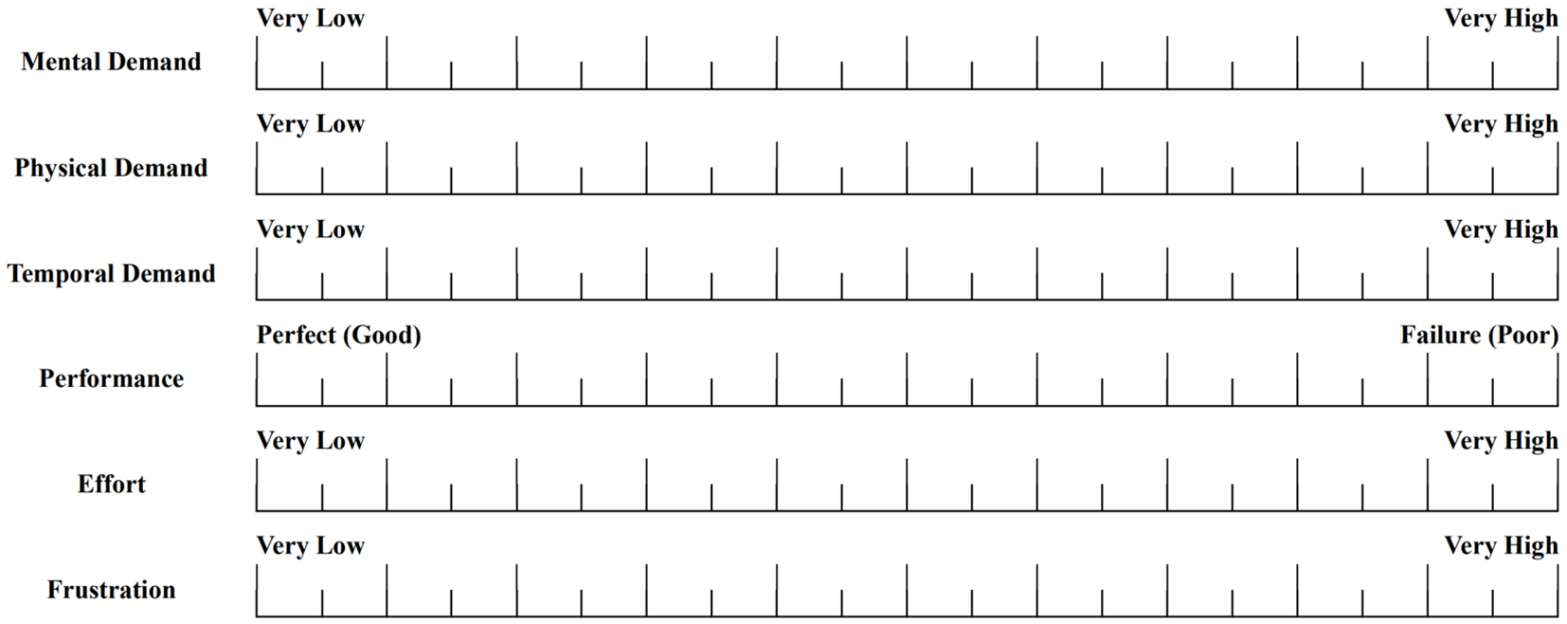
Questions of NASA TLX

The NASA task load index (TLX) is globally used to investigate the subjective workload numerically in experimental tasks, as presented in Fig. 5 and Table 4 [42]. It has six indicators: mental demand, physical demand, temporal demand, performance, effort, and frustration. To calculate the scores of the NASA TLX, weights for the six indicators are computed. Then, scores for each indicator are assigned based on a 21-point Likert scale ranging from 0 (very low, perfect) to + 100 (very high, failure) [42]. The overall score is obtained by multiplying the weights with the scores based on the Likert scale. In this study, a quantitative analysis is conducted using simplified raw NASA TLX scores, thereby simplifying the computation of weights [43].

**Table 4:** Description of NASA TLX

Indicator	Description
Mental demand	How much mental and perceptual activity was required (e.g. thinking, deciding, calculating, remembering, looking, searching, etc.)? Was the task easy or demanding, simple or complex, exacting or forgiving?
Physical demand	How much physical activity was required (e.g. pushing, pulling, turning, controlling, activating, etc.)? Was the task easy or demanding, slow or brisk, slack or strenuous, restful or laborious?
Temporal demand	How much time pressure did you feel due to the rate of pace at which the tasks or task elements occurred? Was the pace slow and leisurely or rapid and frantic?
Performance	How successful do you think you were in accomplishing the goals of the task set by the experimenter (or yourself)? How satisfied were you with your performance in accomplishing these goals?
Effort	How hard did you have to work (mentally and physically) to accomplish your level of performance?
Frustration	How insecure, discouraged, irritated, stressed and annoyed versus secure, gratified, content, relaxed and complacent did you feel during the task?

### Statistical analysis

To compare the values computed for the traditional and proposed methods, the independent T-test, Mann–Whitney U-test, and Welch’s T-test are applied at a 95% confidence level according to the results of the Shapiro–Wilk test for normality and Levene’s test for equality of variance. In detail, if the *p* value derived from the Shapiro–Wilk test is over 0.05, the independent T-test is used; otherwise, the Mann–Whitney U-test is performed as a nonparametric test. In the independent T-test, if the *p* value obtained from Levene’s test is under 0.05, Welch’s T-test is applied (in the Results section, the *p* values obtained from the Mann–Whitney U-test and Welch’s T-test are indicated by single (†) and double (††) dagger symbols, respectively; there is no superscript in the case of the independent T-test). All the statistical analyses are carried out using the statistical package for social sciences (SPSS).

## Results

### Environment validation

The area of the EECS workspace is 4,289.32 cm^3^, which exceeds that of the reference workspace [44]. The communication latencies between the primary and secondary systems from the dVRK is approximately 560 us [28]. The latencies between the SRCI and EECS are under 10 ms. The accuracy of SRCI in recognizing the speech commands when participants perform LTT and SSPT is presented in Table 5. The cases of not recognizing or misinterpreting are included in the failure situation. The accuracies in the novice and surgeon groups in the LTT are 88.35% and 91.01%, respectively and those in the SSPT are 82.11% and 88.54%, respectively. Considering all tasks, the accuracies in the surgeon and novice groups are 90.18% and 86.06%, respectively. The total accuracy calculated from all the cases is 88.07%.

**Table 5:** Accuracy of SRCI

Task type	Group	Total commands	Success	Fail	Accuracy (%)
LTT	Novice	369	326	43	88.35
	Surgeon	378	344	34	91.01
SSPT	Novice	218	179	39	82.11
	Surgeon	192	170	22	88.54

### Task performance

#### LTT

**Fig. 6:**
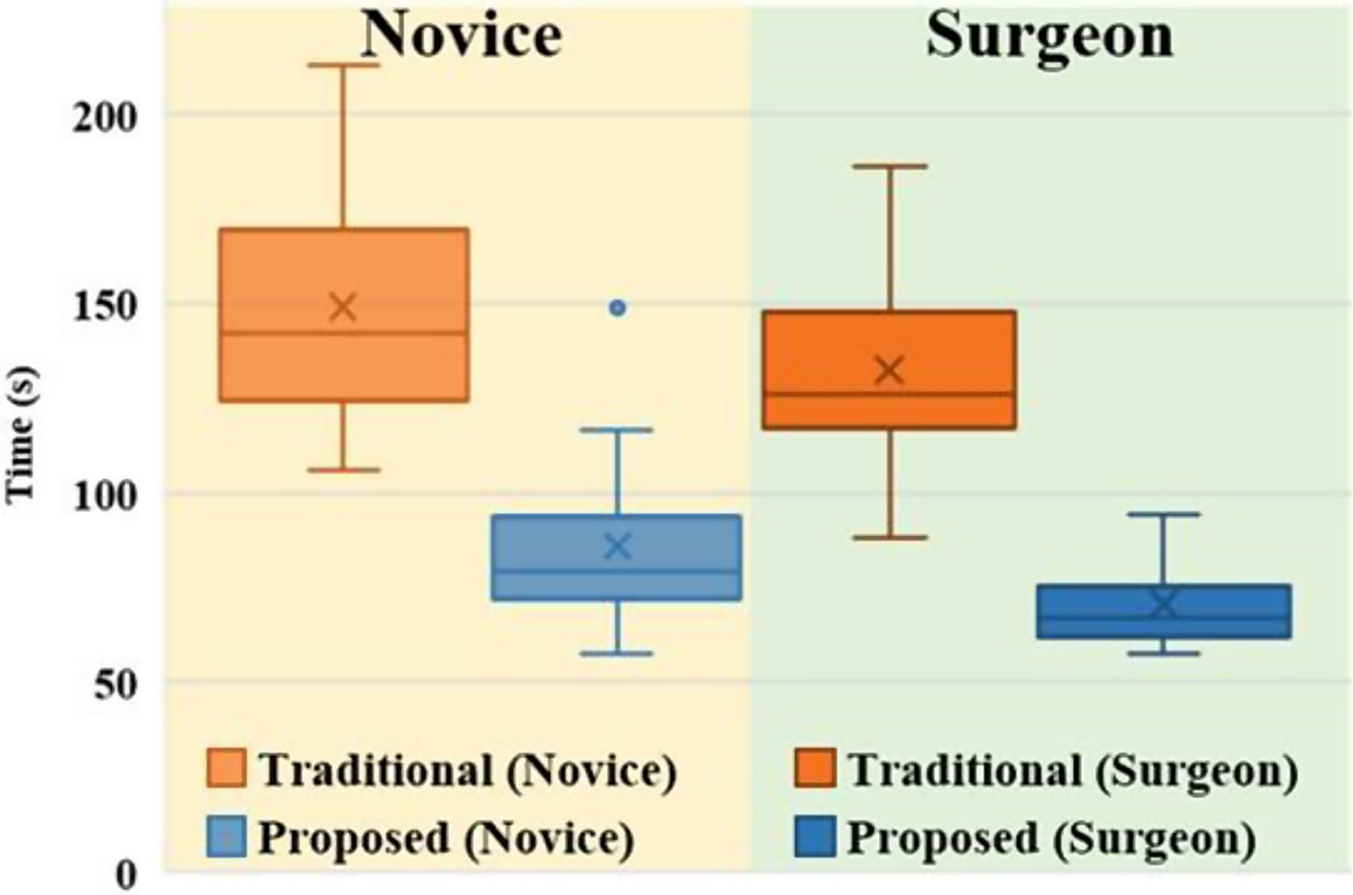
Tendency of completion times in LTT

Figure 6 shows a graph depicting the times required by the different participant groups to complete the LTT using the traditional and proposed methods. The proposed method significantly reduces the LTT completion time compared to the traditional method in both the novice group (from 149.06 s (SD: 30.34 s) to 85.89 s (SD: 22.18 s)) and the surgeon group (from 133.02 s (SD: 26.58 s) to 70.39 s (SD: 10.67 s)) (novice group: *p* < 0.001^†^, surgeon group: *p* < 0.001^†^^†^). The proposed method reduces the completion time by 63.17 s (SD: 24.13 s) in the novice group and by 62.63 s (SD: 23.12 s) in the surgeon group, with no significant difference between the two groups (*p* = 0.944^†^^†^).

#### SSPT

**Fig. 7:**
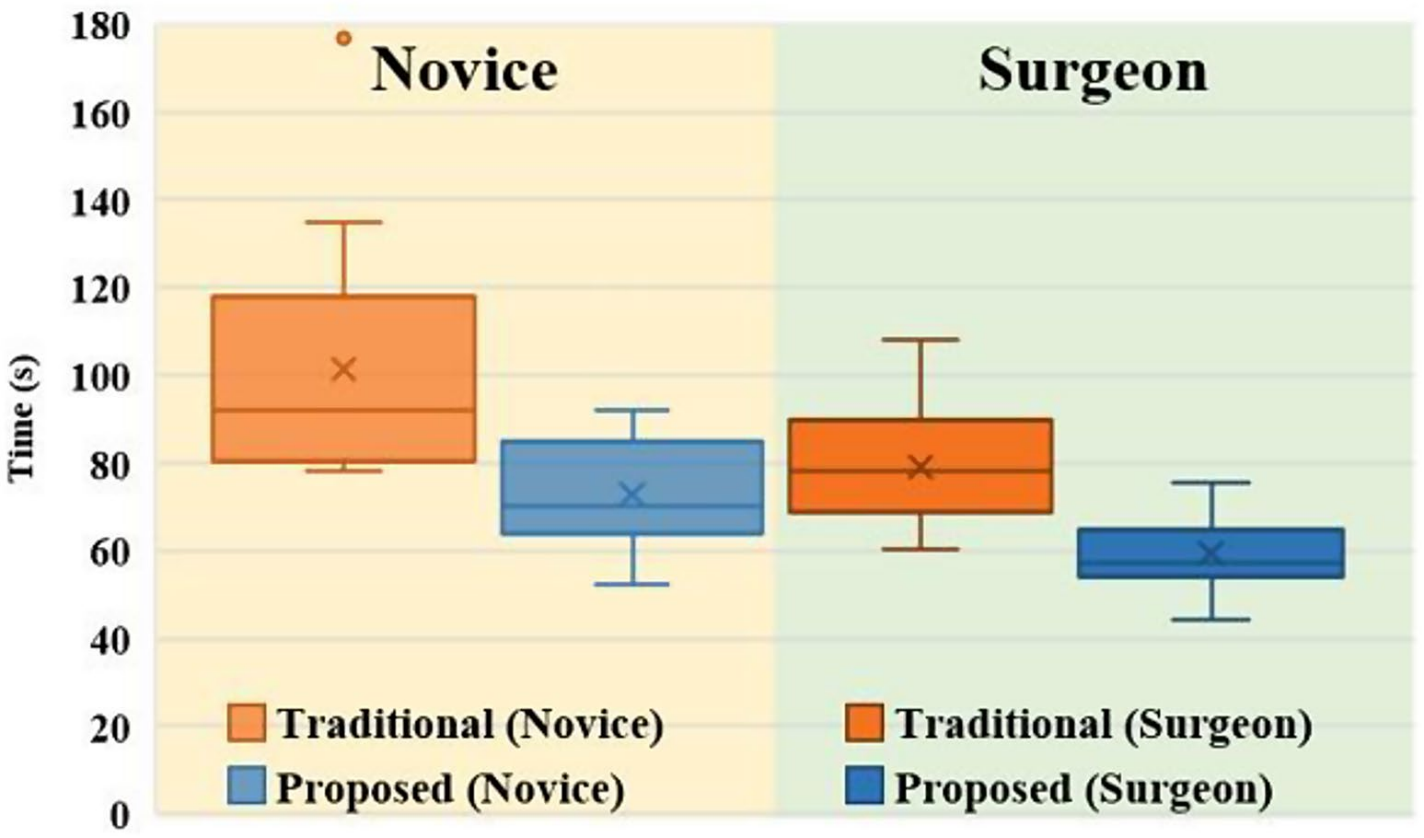
Tendency of completion times in SSPT

Figure 7 shows a graph depicting the times required by the different participant groups to complete the SSPT using the traditional and proposed methods. The proposed method significantly reduces the SSPT completion time compared to the traditional method in both the novice group (from 101.35 s (SD: 25.55 s) to 72.91 s (SD: 11.50 s)) and the surgeon group (from 79.05 s (SD: 13.61 s) to 59.37 s (SD: 8.25 s)) (both, *p* < 0.001^†^^†^). The proposed method reduces the completion time by 28.44 s (SD: 22.80 s) in the novice group and by 19.67 s (SD: 9.99 s) in the surgeon group, with no significant difference in the mean time between the two groups (*p* = 0.146^††^), but with a significant difference in the variances (*p* = 0.024).

### Questionnaires

#### ASQ

The comprehensive results of ASQ are presented in Table 6. The novice participants scored equally for the traditional and proposed methods. In the surgeon group, the mean score for the proposed method is higher than those for the traditional method. For both groups, no statistically significant difference is observed between the results obtained for the traditional and proposed methods.

**Table 6:** Results of ASQ

Group	Mean (SD)		*p* value
	Traditional method	Proposed method	
Novice (*N* = 18)	8.33 (3.48)	8.33 (4.72)	0.535^†^
Surgeon (*N* = 20)	8.95 (4.10)	9.05 (4.10)	0.860^†^
Total (*N* = 38)	8.66 (3.78)	8.71 (4.36)	0.955

#### SUS

**Fig. 8:**
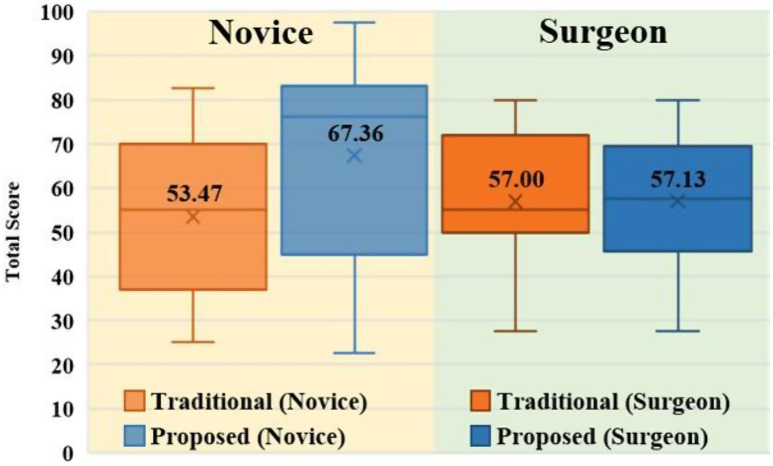
Tendency of comprehensive score in SUS

The comprehensive results of SUS are presented in Table 7. The participants in both groups evaluated the usability of the proposed method to be higher than that of the traditional method. In detail, a statistically significant difference is observed between the results obtained for the novice group, whereas only a slight difference is evident for the surgeon group. Figure 8 demonstrates that all the adjective scores on average belong to the “Good” rating according to the formula calculation [36, 37].

**Table 7:** Results of SUS

Group	Mean (SD)		*p* value
	Traditional method	Proposed method	
Novice (*N* = 18)	53.47 (17.72)	67.36 (22.60)	**0.048**
Surgeon (*N* = 20)	57.00 (16.17)	57.13 (15.07)	0.980
Total (*N* = 38)	55.33 (16.79)	61.97 (19.44)	0.115

#### NASA TLX

The overall results mostly show that the workload in the proposed method is lower than in the traditional method, as represented in Table 8. Especially, the workload indicators, “physical demand” and “effort”, in both groups are less in common in the proposed method than in the traditional method, with statistically significant differences (*p* < 0.05).

**Table 8:** Results of NASA TLX

Indicator	Novice (*N* = 18)			Surgeon (*N* = 20)		
	Mean (SD)		*p* value	Mean (SD)		*p* value
	Traditional method	Proposed method		Traditional method	Proposed method	
Mental demand	54.72 (22.91)	39.72 (21.45)	0.050	44.25 (26.37)	37.25 (25.10)	0.395
Physical demand	48.06 (29.06)	29.44 (22.35)	**0.030** ^**†**^	52.00 (26.43)	23.00 (17.43)	**0.002** ^**†**^
Temporal demand	50.00 (20.72)	46.67 (24.49)	0.662	50.25 (23.14)	54.25 (22.38)	0.582
Performance	42.22 (24.87)	51.11 (24.35)	0.286	37.50 (26.68)	48.25 (23.36)	0.183
Effort	58.61 (22.67)	42.78 (23.21)	**0.045** ^**†**^	53.75 (22.47)	35.00 (20.33)	**0.009**
Frustration	35.83 (26.30)	32.22 (25.62)	0.679	32.75 (20.29)	47.00 (26.53)	0.073^†^
Global score	48.24 (16.13)	40.32 (17.66)	0.169	45.08 (18.51)	40.79 (16.05)	0.438

## Discussion

The present study validates the proposed innovative method that facilitates simultaneous manipulation through speech commands based on comparison with the traditional method using the MTMs. As the preparation step, we focused on designing the simulated operation environment. The motion parameters of the EECS and PSMs were tuned to simulate the manipulators in the dVSS. In the case of the EECS manipulated through the SRCI, the moving ratios of the EECS were also preset based on the scale factor required to control the EECS with the MTM. In surgical robots, though, the fixed velocities of the manipulators cannot be measured because of users’ constantly varying motions in the surgeon console as well as the surgeon’s routine action habits required in their departments. From the perspective of the accuracy of recognizing the speech commands, the results were expected because of the innate differences in the linguistics and phonetics of the Korean and English languages; because of such differences, Korean speakers have difficulty pronouncing certain English words [45]. Besides, the speech recognition model used in the present study has the word error rates in in-domain data and general English are 6.67% and 3.24%, respectively [30]. However, with the advancement of deep learning techniques, next-generation speech recognition is expected to exhibit high performance and become prevalent in various applications including the medical field [46–48]. From the perspective of the SRCI’s latency, it is attributed to interpreting the speech commands by the sequence matching formula that increases the amount of calculation for comparing it based on preset commands in the text [31]. The interpretation speed differs according to the status of Ethernet communication with the Cloud-based application programming interface (API), which can be improved in the future [49].

In LTT and SSPT, the statistically significant difference (*p* < 0.001) in the completion times between the proposed and traditional methods can be attributed to the time required to push the buttons on the foot pedal tray and readjust the position of the MTMs repeatedly in the traditional method. Conversely, continuous manipulation of the PSM and ECM becomes possible through the simple speech commands to the SRCI. As a result, the unnecessary time delay caused by the pedal is reduced in the proposed method. In the SSPT, the more complex task, the novice group showed a greater reduction in completion time using the proposed method, although this reduction was not statistically significant. However, considering the significantly higher variance and the presence of outliers in the novice group, if the group was changed to interns or junior residents with a larger sample size, representing inexperienced surgeons with less variance, the proposed method might demonstrate a significant reduction in completion time compared to the traditional method. In other words, the proposed method may be more beneficial for inexperienced surgeons when performing complex tasks, especially in larger datasets.

The ASQ and SUS results in the surgeon group indicate that the scores for the proposed method are 1.12% and 0.23% higher than those for the traditional method, respectively, with statistically insignificant differences. In NASA TLX, however, the statistical significance of “physical demand” and “effort” indicators in both groups is crucial to alleviating fatigue because physical and cognitive workload is a common phenomenon irrespective of the medical professions of the participants. Moreover, scores for “mental demand” are low for both groups, indicating that confusion due to simultaneous control of the PSM and EECS is less in the proposed method than in the traditional one. Both groups provide low scores for “performance” and “frustration” to the traditional method, which is attributed to issues arising from the latency difference between the traditional and proposed methods. Following the description of “temporal demand” that estimates psychological properties such as time pressure, it can be interpreted that the time reduction of task performance draws the usability enhancement because the indicator can reflect the participants’ subjective feelings while performing the actual task [42]. Even though there were dissimilar results between surgeons and novices in “temporal demand” with insignificant differences, the time reduction of task performance can mean usability improvements, as in the previous study [50].

As a further step, the various feedback collected from the surgeons includes the incorporation of a speed control function in the EECS, the addition of various directions, and increased accuracy of speech recognition as suggestions for potential technological enhancement in the SRCI. With such improvements, SRCI can replace the traditional method using the MTMs. In addition, positive comments are obtained for the convenience of the SRCI, which enables intuitive and continuous movement with a single speech command and does not require repeated actions for controlling the EECS. This feature allows the participants to focus only on manipulating the MTMs to control the surgical instruments without paying attention to controlling the EECS with the MTMs. Besides, no confusion about the location of buttons on the foot pedal tray arises. Considering that the completion time and fatigue encountered during the tasks are reduced, the traditional method with the MTMs can be replaced by the speech-mediated methods that realize simultaneous manipulation for continuous surgical flow.

## Conclusion

The speech-mediated method with the SRCI was proposed to investigate the simultaneous manipulation of the endoscope and surgical instruments in the dVSS. Based on the various usability-focused evaluations, the results demonstrated that the completion times in the LTT and SSPT using the proposed method were less than using the traditional method, with statistically significant differences (*p* < 0.001). The results in ASQ and SUS indicated positive scores for the proposed method, and “physical demand” and “effort” indicators in NASA TLX confirm that such workloads are reduced when using the proposed method. Consequently, the speech-mediated method using the SRCI can replace the traditional method using the MTMs in dVSS, inducing continuous surgical flow during robotic operations.

## Electronic supplementary material

Below is the link to the electronic supplementary material.

Supplementary Material 1
