# Supplementary material for: Speech-mediated manipulation of da Vinci surgical system for continuous surgical flow
Source: Biomed Eng Lett. 2024 Oct 12;15(1):117–29. doi: 10.1007/s13534-024-00429-5 (PMC11704117; doi:10.1007/s13534-024-00429-5)

Supplementary Information

Title

Speech-mediated manipulation of da Vinci surgical system for continuous surgical flow

Author information

The names of authors

Young Gyun Kim[1], Jae Woo Shim[1], Geunwu Gimm[2], Seongjoon Kang[1], Wounsuk Rhee[3], Jong Hyeon Lee[1], Byeong Soo Kim[1], Dan Yoon[1], Myungjoon Kim[4], Minwoo Cho[5,6], and Sungwan Kim[2,7]

The affiliations of the authors

[1] Interdisciplinary Program in Bioengineering, Seoul National University, 1 Gwanak-ro, Gwanak-gu, Seoul, 08826, Republic of Korea

[2] Department of Biomedical Engineering, Seoul National University College of Medicine, 103 Daehak-ro, Jongno-gu, Seoul, 03080, Republic of Korea

[3] Seoul National University Hospital, 101 Daehak-ro, Jongno-gu, Seoul, 03080, Republic of Korea

[4] MedInTech Inc., 60 Daehak-ro, Jongno-gu, Seoul, 03100, Republic of Korea

[5] Department of Transdisciplinary Medicine, Seoul National University Hospital, 101 Daehak-ro, Jongno-gu, Seoul, 03080, Republic of Korea

[6] Department of Medicine, Seoul National University College of Medicine, 103 Daehak-ro, Jongno-gu, Seoul, 03080, Republic of Korea

[7] Artificial Intelligence Institute, Seoul National University, 1 Gwanak-ro, Gwanak-gu, Seoul, 08826, Republic of Korea

Young Gyun Kim, Jae Woo Shim, and Geunwu Gimm are co-first authors and contributed equally to this work.

Minwoo Cho and Sungwan Kim are co-corresponding authors.

E-mail addresses of the corresponding authors

Minwoo Cho: chovis@snuh.org
Sungwan Kim: sungwan@snu.ac.kr

ORCID of the authors

Young Gyun Kim: 0000-0003-1231-9097
Jae Woo Shim: 0000-0002-3940-1309
Genuwu Gimm: 0000-0002-7414-4125
Seongjoon Kang: 0009-0004-4923-741X
Wounsuk Rhee: 0000-0001-7770-5760
Jong Hyeon Lee: 0000-0002-1989-5997
Byeong Soo Kim: 0000-0001-8767-9842
Dan Yoon: 0000-0002-5657-5984
Myungjoon Kim: 0000-0002-4176-546X
Minwoo Cho: 0000-0003-2242-4747
Sungwan Kim: 0000-0002-9318-849X

Table 6 ASQ (global reliability: 0.96)

No.	Question	Score*						
		1	2	3	4	5	6	7
1	Overall, I am satisfied with the ease of completing the tasks in this scenario.	○	○	○	○	○	○	○
2	Overall, I am satisfied with the amount of time it took to complete the tasks in this scenario.	○	○	○	○	○	○	○
3	Overall, I am satisfied with the support information when completing the tasks.	○	○	○	○	○	○	○

*Seven-point Likert scale ranging from +1 (strongly agree) to + 7 (strongly disagree)

Table 7 SUS (global reliability: 0.92)

No.	Question	Score*				
		1	2	3	4	5
1	I think that I would like to use this system frequently.	○	○	○	○	○
2	I found the system unnecessarily complex.	○	○	○	○	○
3	I thought the system was easy to use.	○	○	○	○	○
4	I think that I would need the support of a technical person to be able to this system.	○	○	○	○	○
5	I found the various functions in this system were well integrated.	○	○	○	○	○
6	I thought there was too much inconsistency in this system.	○	○	○	○	○
7	I would imagine that most people would learn to use this system very quickly.	○	○	○	○	○
8	I found the system very awkward to use.	○	○	○	○	○
9	I felt very confident using the system.	○	○	○	○	○
10	I needed to learn a lot of things before I could get going with the system.	○	○	○	○	○

*Five-point Likert scale ranging from +1 (strongly disagree) to +5 (strongly agree)

Table 8 Description of NASA TLX

Indicator	Description
Mental demand	How much mental and perceptual activity was required (e.g. thinking, deciding, calculating, remembering, looking, searching, etc.)? Was the task easy or demanding, simple or complex, exacting or forgiving?
Physical demand	How much physical activity was required (e.g. pushing, pulling, turning, controlling, activating, etc.)? Was the task easy or demanding, slow or brisk, slack or strenuous, restful or laborious?
Temporal demand	How much time pressure did you feel due to the rate of pace at which the tasks or task elements occurred? Was the pace slow and leisurely or rapid and frantic?
Performance	How successful do you think you were in accomplishing the goals of the task set by the experimenter (or yourself)? How satisfied were you with your performance in accomplishing these goals?
Effort	How hard did you have to work (mentally and physically) to accomplish your level of performance?
Frustration	How insecure, discouraged, irritated, stressed and annoyed versus secure, gratified, content, relaxed and complacent did you feel during the task?

Fig. 8 Questions of NASA TLX

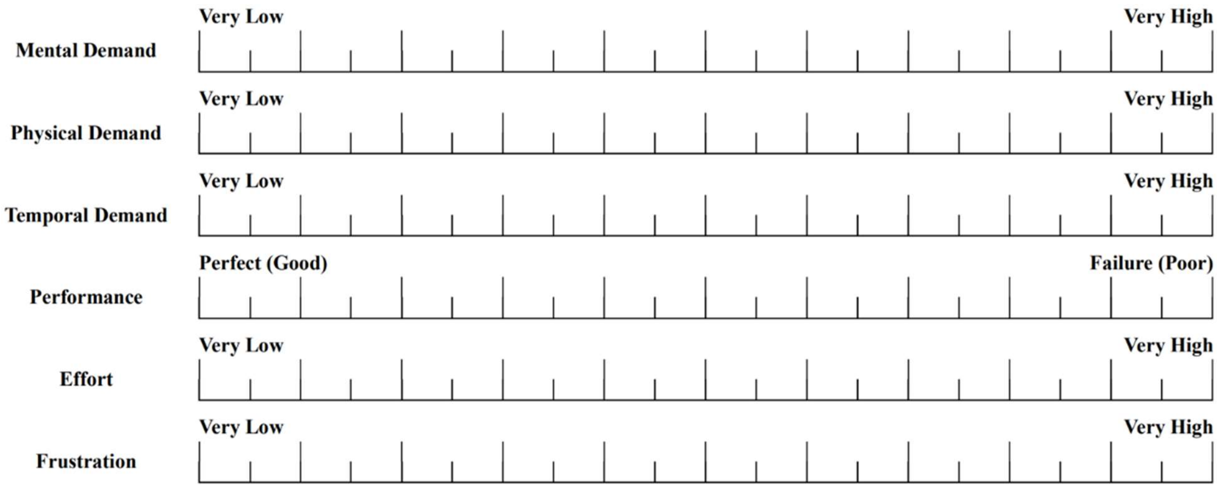

Supplement: Supplementary file 1 — Supplementary Material 1 [file 13534_2024_429_MOESM1_ESM.pdf]
